# Double-edged sword of diabetes mellitus for abdominal aortic aneurysm

**DOI:** 10.3389/fendo.2022.1095608

**Published:** 2022-12-16

**Authors:** Zijia Huang, Huiling Su, Tiejun Zhang, Yuwen Li

**Affiliations:** ^1^ Department of Pharmacy, West China Hospital, Sichuan University, Chengdu, Sichuan, China; ^2^ Department of Neurosurgery, West China Hospital, Sichuan University, Chengdu, Sichuan, China

**Keywords:** abdominal aortic aneurysm, diabetes mellitus, hyperglycemia, mortality, rupture

## Abstract

**Introduction:**

Diabetes mellitus (DM) has been proved to contribute to multiple comorbidities that are risk factors for abdominal aortic aneurysm (AAA). Remarkably, evidences from epidemiologic studies have demonstrated a negative association between the two disease states. On the other hand, hyperglycemic state was linked to post-operative morbidities following AAA repair. This review aims to provide a thorough picture on the double-edged nature of DM and major hypoglycemic medications on prevalence, growth rate and rupture of AAA, as well as DM-associated prognosis post AAA repair.

**Methods:**

We performed a comprehensive search in electronic databases to look for literatures demonstrating the association between DM and AAA. The primary focus of the literature search was on the impact of DM on the morbidity, enlargement and rupture rate, as well as post-operative complications of AAA. The role of antidiabetic medications was also explored.

**Results:**

Retrospective epidemiological studies and large database researches associated the presence of DM with decreased prevalence, slower expansion and limited rupture rate of AAA. Major hypoglycemic drugs exert similar protective effect as DM against AAA by targeting pathological hallmarks involved in AAA formation and progression, which were demonstrated predominantly by animal studies. Nevertheless, presence of DM or postoperative hyperglycemia was linked to poorer short-term and long-term prognosis, primarily due to greater risk of infection, longer duration of hospital stays and death.

**Conclusion:**

While DM is a positive factor in the formation and progression of AAA, it is also associated with higher risk of negative outcomes following AAA repair. Concomitant use of antidiabetic medications may contribute to the protective mechanism of DM in AAA, but further studies are still warranted to explore their role following AAA repair.

## 1 Introduction

Abdominal aortic aneurysm (AAA) is defined as an abnormal focal expansion of the abdominal aorta with a diameter greater than 3 cm or 50% larger than its normal size (1). AAA is one of the main vascular complications that pose a rising health burden worldwide (2) especially in low-income and middle-income countries due to aging population, increase in cigarette smoking and rising challenges of chronic comorbidities (3). Patients with AAA carries a high risk of mortality due to rupture, and current intervention for AAA relies exclusively on surveillance or surgical repair (4), which underlines the need to identify effective pharmacological target.

Important risk factors contributing to AAA comprise advanced age, male gender, smoking, as well as comorbidities such as history of other vascular complications, coronary artery disease, atherosclerosis, hypercholesterolemia, and hypertension (1). Diabetes mellitus (DM) is a major risk factor to atherosclerotic lesions and macrovascular diseases that are linked to AAA presence, including cardiovascular disease (CAD) and peripheral vascular disease (PAD) (5). Surprisingly, instead of accelerating the formation and progression of AAA, DM was found to have an inverse association with AAA development in previous epidemiological reports (6, 7). DM exerts a protective effect potentially by suppressing major pathological hallmarks of AAA (4, 8).

On the other hand, DM is a known risk factor for higher perioperative or postoperative morbidity and mortality. Post-operative hyperglycemia is associated with delayed would healing and higher infectious complication. Therefore, the protective effect of DM to AAA might be attenuated following AAA repair. This study aims to provide insight to standard of care for patients with both AAA and DM by reviewing the paradoxical effects of DM and hypoglycemic agents on prevalence, growth rate and rupture of AAA, as well as DM-associated morbidity and mortality post AAA operations.

## 2 Features of AAA pathogenesis targeted by DM

The mechanism underlying the pathogenesis of AAA was found related to the biology of abdominal aortic wall. Diabetic patients were shown to have decreased levels of matrix metalloproteinases (MMPs) and matrix degradation, enhanced matrix and collagen synthesis, resulting in a thicker abdominal aortic wall and a larger volume of extracellular matrix (ECM) (7, 9, 10).

ECMs in AAA patients are characterized by extensive proteolysis, leading to the destruction of collagen and elastin. Diabetes mellitus induces glycation of the ECM, which subsequently stimulates the formation of advanced glycation end products (AGE) (7). AGEs are covalently cross-linked with elastin and collagen in the blood vessel wall, promoting protection against mechanical structure loss and contributing to arterial stiffness (7, 9, 11, 12). Although enhanced arterial stiffness is a risk factor for atherosclerotic diseases, it provides resistance towards aneurysm growth delays AAA progression (12, 13). Nevertheless, the influence of AGEs on AAA was found conflicting. Stimulation of the AGEs receptor (RAGE) leads to upregulation of inflammatory cytokines and MMPs, thus promoting the formation of AAA (7, 14, 15). Through a murine elastase-induced AAA model, Raaz et al. discovered that segmental aortic stiffening enhanced aortic wall stress and promoted aneurysmal growth. In contrast, homogenous stiffening reduced aneurysm growth (12, 16).

MMPs are calcium-dependent zinc endopeptidases secreted by vascular endothelial cells and macrophages; they play a key role in the pathogenesis of AAA. Both MMP-2 (released by smooth muscle) and MMP-9 (released by macrophages) were found involved in the process of matrix destruction and vessel wall degradation in aortic aneurysms (9). Expression of MMP-9 was observed elevated at the site of AAA rupture, and was additionally associated with ruptured aneurysm related 30-day mortality (17, 18). The expressions of pro-MMPs and MMPs in diabetic patients are significantly attenuated. Hyperglycemic state inhibits the expression of MMP-9 messenger RNA and protein expression in macrophage cell lines by stimulating glucose sensitive nuclear receptor Nr1h2 (19), which may explain the aortic wall thickening and matrix loss deceleration in diabetic aneurysms (11).

In addition, inflammatory processes exert a significant influence on aortic wall remodeling in AAA pathogenesis. The underlying mechanisms by which diabetes affects inflammatory process may include activation of T cell insulin receptors, the monocyte-macrophage system, and through C-peptides production (9, 14). DM patients often have elevated circulating C-peptide and macrophage levels in aortic tissues. A previous study demonstrated that C-peptide impairs high glucose-induced proliferation and nuclear factor kappa B (NF-κB) nuclear translocation in vascular smooth muscle cells (VSMCs) (20). In the presence of C-peptide, the expression of various pro-inflammatory cytokines is reduced *via* the NF-κB pathway (9, 14).

Homeostasis of VSMC is another protective factor towards AAA. In most cases, VSMC exists in contractile phenotype, which contribute to vascular remodeling (1, 9). When exposed to oxidative stress, inflammation or injury, VSMC of contractile phenotype will differentiate into a synthetic phenotype. Synthetic VSMC phenotype is implicated with decreased expression of contractile protein, increased MMPs and vascular calcification, thereby potentiating AAA progression and rupture (9, 21, 22). Transforming growth factor (TGF)-β was found essential for the induction and maintenance of environmental balance and differentiation in VSMC. Hyperglycemic state triggers TGF-β signaling pathway, thereby downregulating the expression of MMP-2 and exerting protective effect on aortic VSMC (14, 23). Moreover, an *in vitro* study discovered significant morphological differences between Type II DM (T2DM) and non-T2DM VSMC. The study found vinculin, a local adhesive protein that couples the ECM to the cytoskeleton, was upregulated in T2DM VSMC (24). Increased vinculin-positive focal adhesions in VSMC may account for the vascular stiffness of the abdominal aortic wall in DM patients, leading to retard of AAA formation (7, 10).

Intraluminal thrombus (ILT) is another increasingly recognized feature of AAA growth, remodeling, and rupture over the past decades (25). Tissues of AAA subjects were found with greater concentrations of tissue plasminogen activator (TPA) and hypoactive plasminogen activator inhibitor (PAI-1), implying a hypercoagulable state that stimulates ILT deposition and reduces ILT renewal (26). ILT is implicated with cellular inflammation, arterial wall hypoxia and ECM apoptosis, prompting aneurysm growth and eventual rupture (25). In addition, plasmin converts pro-MMPs to their active form; by inhibiting conversion of plasminogen to plasmin, PAI-1 attenuates fibrinolysis and decreases MMP production. Downregulation of PAI-1 in AAA tissues thereby results in augmented MMPs production in ILT and in the AAA wall (27–29). Dua et al. investigated the effect of hyperglycemia on endogenous PAI-1 in AAA-induced mice. Immunohistochemistry confirmed increased intensity of PAI-1 and suppressed MMP-9 expression in the diabetic mice, corresponding to a reduced AAA formation (30).

**Figure 1:**
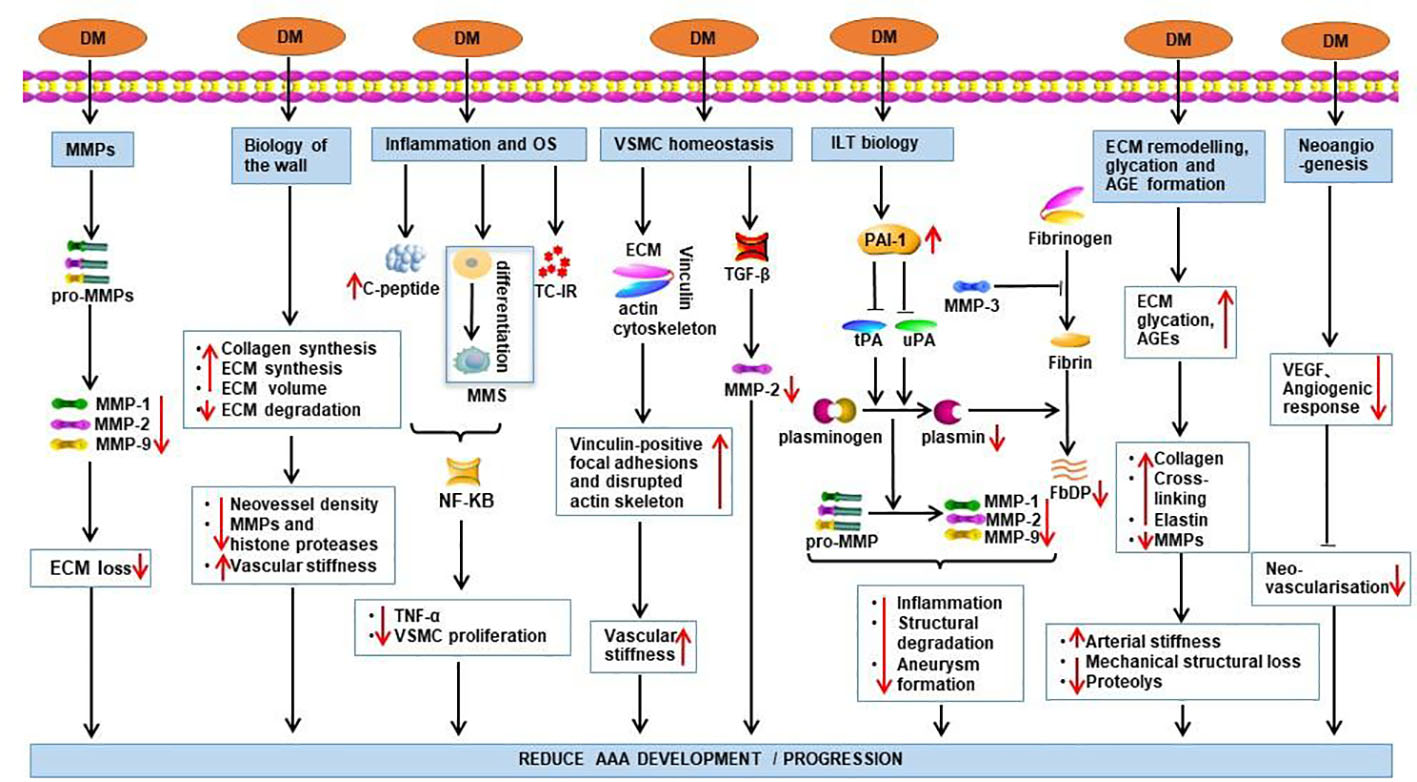
Potential mechanisms by which DM exerts its protective effect on AAA. Effects of DM are demonstrated by red arrows. TC-IR, T-cell insulin receptors; DM, diabetes mellitus; MMS, monocyte-macrophage system; OS, oxidative stress; FbDP, Fibrin degradation products; VSMC, Vascular smooth muscle cells; ILT, intraluminal thrombus; PAI-1, Plasminogen activator inhibitor-1.

Moreover, thicker regions of ILT are linked to localized hypoxia in AAA, subsequently induces a variety of angiogenesis control factors such as vascular endothelial growth factor (VEGF); these changes induce localized mural neovascularization and inflammation, in addition to regional wall weakening (31). Hyperglycemia inhibits neovascularization by downregulating the expression of VEGF and angiogenesis response (9, 32). The protective mechanisms of DM against AAA are summarized in **Figure 1**.

## 3 Protective effect of DM to AAA

### 3.1 Effect on prevalence rate

AAA is most commonly diagnosed in male among the age of 60 to 80 (33). Currently known risk factors for AAA include male, smoking, family history, advancing age, hypertension, as well as obesity (34). While most of the risk factors are also linked to DM, previous meta-analysis and epidemiological studies identified DM diagnosis as a negative predictor for AAA (35, 36). The presence, growth, and probably rupture of AAA were found to be higher among non-diabetics comparing to diabetic patients (6, 7).

The negative association between DM and AAA was also demonstrated by pooled data analysis from large population prevalence studies and smaller studies in selected populations. Prospective studies showed significantly lower number of new AAA diagnosis in DM patients (37). When diabetic patients were stratified into advanced and uncomplicated groups based on existing DM comorbidities, it was demonstrated that advanced diabetes exerts a stronger protection against AAA without rupture, with risk of AAA decreased by near a half in the advanced DM group (6). Le et al. also revealed a decreasing risk of AAA with longer diabetes duration in addition to the independent inverse association between DM and AAA. The odds ratio of AAA was 0.50 at 3–5 years of follow-up (95% CI 0.27, 0.89), which declines further to 0.37 after 12 years of follow-up (95% CI 0.19, 0.70) (38). This outcome was compatible to the conclusion of another study, which discovered no difference in aortic diameter and AAA prevalence between those newly diagnosed type 2 diabetes and those without diabetes among 65-year-old men (39). In addition, an inverse association was established between fasting serum glucose level and aortic diameter even among non-diabetic men (38).

### 3.2 Effect on growth rate

Rabben et al. identified 4 independent AAA risk factors: smoking, hypertension, BMI >30, and DM, with DM being an inverse factor for AAA. The growth rate of AAA was slower in DM patients comparing to normoglycemic patients (34). Astrand et al. found the aortic intima-media thickness (IMT) to be markedly greater in diabetic patients comparing to healthy controls, adjusting for age and sex. The thick aortic wall in diabetes resulted in a 20% reduction in aortic wall stress, which proposed a potential protective mechanism against AAA (40). Another multicenter randomized study enrolling patients with small AAAs (4.1-5.4 cm) revealed a remarkably lower probability of aneurysm growth > 5 mm at 36 months in diabetic participants comparing to nondiabetics (40.8% versus 85.1%). For patients who did not undergo open repair at baseline, the need for repair at 30 months was also lower for diabetics. However, this study also demonstrated a higher hazard ratio (HR) for all-cause mortality at 36 months among diabetic patients, which could be attributed to higher obesity and cardiovascular disease rate (41).

A sub-analysis study of VIVA (Viborg Vascular) Randomized Screening Trial found that the median growth rate significantly slower in subjects with DM comparing to those without (1.7 versus 2.7 mm/year). When participants were stratified by glycosylated hemoglobin (HbA1C), the aorta growth was smaller in the highest HbA1c-tertile group compared with the lowest HbA1c-tertile group, adjusting for covariates (42).

### 3.3 Effect to RAAA

Despite advancements in surgical technologies, RAAA is still the major cause of AAA-related death, resulting in approximately ninety percent of mortality rate (33). The impact of DM on development and outcome of RAAA is controversial. Overall, there are three conflicting theories regarding the relationship between DM and RAAA: no association, negative association and positive association.

A large Danish register based matched case control study by Kristensen et al. (35) included 5395 cases with ruptured AAA (RAAA) matched 1:1 with patients undergoing elective AAA repairs by sex, age, and year of diagnosis. Outcomes of the study indicated that presence of DM did not protect against the risk of RAAA or influence overall 30-day mortality. A retrospective multivariate analysis study by Gokani et al. also found no statistically significant association between DM status and risk of aneurysm rupture, adjusting for cofactors (43).

In some other studies, DM is reported to be reversely associated with RAAA. A nationwide multicenter study in France reported a significantly lower prevalence of DM in ruptured AAA comparing to non-ruptured AAA (44). However, this study also demonstrated no significant difference between DM and non-DM patients in terms of in-hospital mortality in the ruptured AAA cohort. A meta-analysis by Takagi et al. also described significantly lower prevalence/incidence of RAAA in diabetic patients (odds ratio/hazard ratio, 0.71; 95% Confidence Interval, 0.56 to 0.89; p=0.003) (45). Theivacumar et al. investigated the relationship between DM and any aortic aneurysm rupture at a single center, and concluded that aortic aneurysm rupture and aneurysm rupture-related death are less likely to occur among diabetic patients (46).

On the other hand, epidemiological research of the National Health Fund and the Central Statistical Office in Poland in 2012 found significantly higher incidence of RAAA in diabetic population, regardless of gender stratification (47). Regrettably, data limitations inhibited the evaluation of the type or duration of diabetes, as well as the relationship of RAAA with the presence of cofounding factors such as hypertension, cigarette smoking and lipid values. Based on the paradoxical research outcomes regarding this issue, further prospective epidemiological research with standardized methodology is warranted to determine the link between DM and RAAA.

### 3.4 Gender-specific effect of DM

Previous studies have demonstrated significantly lower AAA incidence and rupture rate in women comparing to men (47). Intriguingly, the protective effect of diabetes was found attenuated among female patients. In a population-based data analysis, the sex-specific analysis showed no difference in AAA incidence rates between DM and non-DM cohorts for females, whereas the incidence rate was significant lower in males with type 2 DM (6). Another subgroup analysis of a register-based matched case control study also found increased risk of AAA rupture in female patients (35). Tsai et al. hypothesized that the diminished protective effect of DM in women was prompted by higher oestrogen level (6), which needs further investment based on paradoxical evidences in previous animal (48, 49) and clinical studies (50, 51).

## 4 Effect of hypoglycemic medications to AAA

Diabetes counteracts formation and progression of AAA; therefore, it has been hypothesized that antidiabetic medicines may reverse the protective role of diabetes against AAA. Intriguingly, both animal and clinical studies have demonstrated protective effect of antidiabetic medications such as metformin, thiazolidinedione, and dipeptidyl peptidase 4 inhibitors (DPP-4i) against aortic aneurysms (14, 52). These major antidiabetics may help to reduce the prevalence, incidence and enlargement rate of AAA (53, 54) in a dose-response pattern (11, 55).

### 4.1 Metformin

Metformin is the first-line oral antidiabetic medicine for mild to moderate type 2 DM patients. It is also the most well-studied hypoglycemic drug in AAA. Previous studies illustrated the negative association between metformin and development of AAA. Metformin was shown to retard the formation and growth rate of AAA (56), even in normoglycemic mice (52, 57). Golledge et al. revealed that patients with diabetes prescribed metformin were associated with significantly lower AAA repair and rupture-related mortality comparing to patients with diabetes not prescribed metformin or patients with no diabetes. In contrary, diabetic patients not prescribed metformin did not show reduced incidence of AAA events comparing to patients with no diabetes (58), which raised the question that whether the protective effect of DM comes from anti-diabetic medicines. Itoga et al. found that patients with a metformin prescription had a 0.20 mm reduction in yearly AAA enlargement comparing to those without metformin prescription, adjusting for other AAA-related variables (59). While it was hypothesized that by attenuating arterial accumulation of matrix molecules, metformin could reverse the protective mechanism of diabetes and lead to increased AAA incidences, Kristensen et al. demonstrated a statistically nonsignificant protective effect of long-term metformin against ruptured AAA (RAAA) (55).

**Figure 2:**
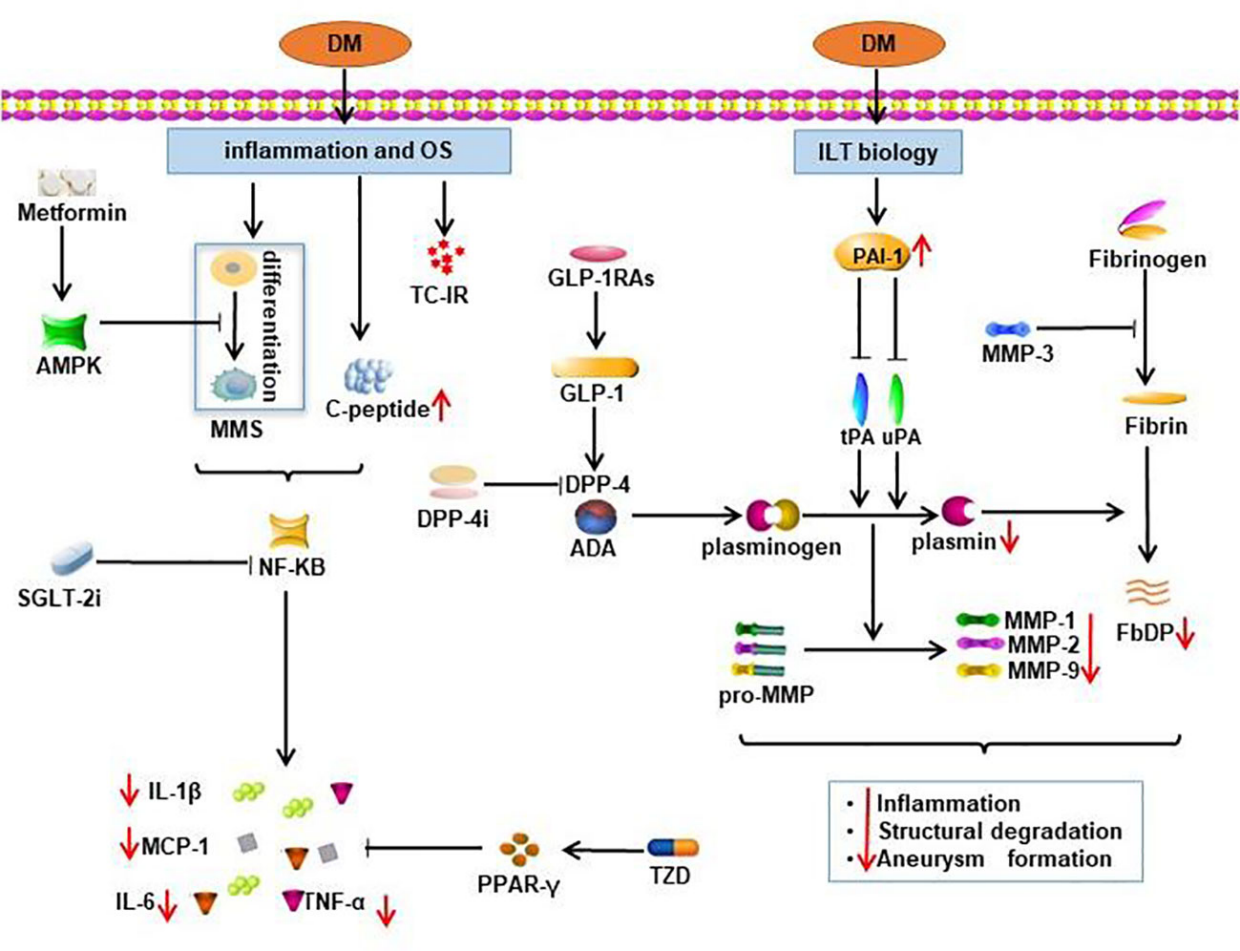
Potential mechanisms of the protective effect of oral antidiabetics on AAA. Effects of oral antidiabetics are demonstrated by red arrows. TC-IR, T-cell insulin receptors; DM, diabetes mellitus; MMS, monocyte-macrophage system; OS, oxidative stress; FbDP, Fibrin degradation products; DPP-4i, dipeptidyl peptidase 4 inhibitors; SGLT-2i, sodium-glucose cotransporter 2 inhibitors; GLP-1RAs, glucagon-like peptide 1 receptor agonists.

Studies mentioned above showed the promising clinical effect of metformin in limiting AAA progression. Utilization of metformin markedly reduced the maximum aortic diameter and formation of aortic aneurysm (14). Metformin activates the AMPK signal pathway, thereby downregulating the expression of proinflammatory cytokines (IL (interleukin)-1β, IL-6, MCP (monocyte chemotactic protein)-1 and TNF (tumor necrosis factor)-α), vascular growth cytokines (VEGFA, Flt-1 and CD31) and MMPs (60). The underlying mechanisms of metformin in AAA pathology eventually lead to preservation of smooth muscle cell (SMC), suppression of matrix remodeling and inhibition of inflammation pathways (61, 62). Treatment with metformin in normoglycemic mice also showed suppression of AAA formation and progression *via* restoration of the perivascular adipose tissue (PVAT) and vascular endothelial function (63). Some other potential biological pathways involved in the protective mechanism of metformin against AAA include: reduction of ECM volume, augmented arterial wall matrix formation through advanced glycation end products (8) and suppression of inflammation and oxidative stress (14). The potential mechanisms of metformin are illustrated in **Figure 2**.

Metformin is excreted through kidney, thereby clearance of metformin decreases during acute or chronic renal function impairment. Given the risk of contrast-induced nephropathy (CIN) after endovascular aneurysm repair (EVAR) (64) and the risk of metformin-induced lactic acidosis in patients with renal insufficiency (65), perioperative administration of metformin should be cautiously monitored. The stop and reinitiating criteria of metformin as recommended by Society for Vascular Surgery (SVS) practice guideline is presented in **Table 1**.

**Table 1:** Association between renal function status and duration of metformin administration.

eGFR(mL/min)	Cessation of Metformin	Re-initiation of Metformin
<60	At the time of contrast administration	No sooner than 48 hours after contrast administration (if renal function remains stable)
<45	Up to 48 hours before contrast administration	

### 4.2 Thiazolidinedione

TZD hypoglycemic agents (such as pioglitazone and rosiglitazone) were found to active peroxisome proliferator-activated receptor-γ (PPARγ). In animal studies, PPARγ was proved to balance the aortic inflammatory conditions and to delay the progression and rupture of AAA (66). TZDs are PPARγ agonists that inhibit inflammatory response and ECM remodeling, thereby ameliorating AAA development and rupture in angiotensin II (Ang II)-induced mouse model (67–70).

Que et al. discovered that pioglitazone activates PPARγ and antagonizes the nuclear factor of activated T-lymphocytes (NFAT)/NF-κB, thus decreasing the protein expression of SMC phenotypic modulation markers. Downregulation of these modulation markers prevents cell proliferation, migration, and macrophage adhesion to SMCs, which ameliorates angiotensin II-induced aortic aneurysms (70). The expression of PPARγ in bone marrow mesenchymal stem cells of AAA patients was found notably upregulated after the administration of pioglitazone (71). The role of pioglitazone is also related to the reduction of macrophages infiltration in aortic wall and retroperitoneal periaortic fat. In a study involving sixteen patients with AA (> 5 cm in diameter) awaiting open surgical repair (OSR), after 2 months of pioglitazone pretreatment, the macrophages infiltration was diminished (72).

PPARγ activation by rosiglitazone was also reported to reduce the availability of inflammatory mediators, thereby influencing aneurysm formation and AT1a Ang II receptor expression (66, 67). Rosiglitazone can reduce the expression of TNF-α and MMP-9 in abdominal aortic aneurysm wall and retroperitoneal abdominal aortic fat (66). By increasing the production of collagen, rosiglitazone also leads to thickening of the aortic wall to reduce aortic dilation and late aneurysm rupture (66). Moreover, rosiglitazone also plays a potential protective role in the formation and rupture of aneurysms through the inhibitory effect on c-Jun N-terminal kinase (JNK) phosphorylation and toll-like receptor 4 (TLR4) expression at the site of lesion formation (68).

### 4.3 Dipeptidyl peptidase 4 inhibitors

DPP-4 is a glycoprotein involved in cleavage and inactivation of a variety of substrates, including incretins such as glucagon-like peptide1 (GLP-1) (4). The expression of DPP-4 in AAA media and adventitia is positively correlated with typical aneurysmal disease processes, including numerous immune responses, ECM degradation and peptidase activity, angiogenesis and reactive oxygen species (73). DPP-4 also increases plasmin levels by binding to adenosine deaminase, which results in degradation of ECM and activation of MMPs *via* activation of plasminogen-2 (74). Moreover, DPP-4 and the DPP-4-like enzyme attractin were found to induce inflammation cascades that are critical to AAA development (4).

DPP-4 inhibitors (such as sitagliptin, alogliptin and linagliptin) delay GLP-1 degradation *via* DPP-4 activity suppression, thereby improving glucose control (11). DPP-4Is also antagonize functions of DPP-4 in previous mentioned AAA pathological pathways, which consequently reduces the formation and development of AAA (4, 74, 75). in a rat-based AAA model by Bao et al., alogliptin administrations in both low-does (1 mg/kg/d) and high-dose (3 mg/kg/d) groups were associated with significant reduction of reactive oxygen species (ROS) expression comparing to the control group on day 7. However, the effect became only significant in high-dose group on day 28. All the other observed effects such as decreased MMP level and dilation in aortic aneurysm wall were also more prominent in the high-dose group. This dose-dependent pattern could be further explored in clinical study (76). Lu et al. investigated the function of another DPP-4I sitagliptin in Ang II-infused mice. Their results demonstrated that sitagliptin may attenuate AAA formation by restraining microphage filtration, MMPs production as well as elastin destruction (77). Other DPP4-Is, teneligliptin and vildagliptin also showed similar protection mechanisms in AAA formation and development (4, 78).

### 4.4 Glucagon-like peptide 1 receptor agonists

GLP-1 is the main insulin-stimulating hormone that induces insulin secretion after nutrient intake. It involves in islet beta cell proliferation and survival, glucagon secretion regulation, and gastrointestinal motility control, leading to the development of effective pharmacological treatment against DM and obesity (75). The positive effects of GLP-1RAs on AAA formation is similar to DPP-4Is, as they share same targets in pathological pathways of AAA (75).

In animal-based AAA models, GLP-1RAs (such as liraglutide and lixisenatide) inhibit AAA development through ECM preservation and through antioxidant and anti-inflammatory effects (75). Lixisenatide attenuates ROS expression and oxidative DNA damage, which in turn retards the inflammatory process of macrophage filtration, pro-inflammatory cytokine release, and eventual MMPs expression (79). Lu et al. also proved that liraglutide reduced Ang II-treated ROS production in U937 human mononuclear cell lines, confirming its antioxidative effect (77). Lixisenatide was also found to decrease levels of extracellular signal-regulated kinase (ERK), which plays a crucial role in the regulation of MMP secretion, thereby ameliorates aortic dilatation (79).

### 4.5 Sodium-glucose cotransporter 2 inhibitors

SGLT-2I is a novel class of hypoglycemic agent that has been investigated beyond its anti-glycemic indication. SGLT-2Is, such as dapagliflozin and empagliflozin, were found to improve the cardiovascular outcomes in patients with heart failure. In an Ang II-induced dissecting AAA mouse model, cotreatment with empagliflozin resulted in significant reduction in maximal suprarenal aortic diameter. Immunohistochemistry study further confirmed that empagliflozin disrupted elastin degradation, neovascularization, and macrophage infiltration in the AAA formation process (80). p38 mitogen-activated protein kinase (p38 MAPK) was previously proved to promote MMP production, while nuclear factor-κB (NF-κB) was known to upregulate cytokines/chemokines in the vascular wall, leading to AAA growth. Empagliflozin blunted p38 MAPK and NF-κB phosphorylation, thereby restricting AAA growth (80). Another study discovered that dapagliflozin markedly impairs medial SMC loss and alleviated aneurysmal aortic expansion in mice treated with intra-aortic porcine pancreatic elastase (PPE) infusion (54).

## 5 Effects of hyperglycemia on morbidity and mortality post AAA repair

Although DM and hypoglycemia medications are reported to have a protective effect against formation and progression of AAA, the role of hyperglycemia in post AAA repair might not be as beneficial. Inadequately controlled blood glucose is known to result in worse operative outcomes, with a higher incidence of infections, delayed wound healing, as well as increased mortality and length of hospital stays. Raffort et al. reported significantly higher total in-hospital mortality among DM patients with unruptured AAA, regardless of open or endovascular repair in a retrospective nationwide multicenter study (44). In addition, survival analysis identified a worse post-operative long-term mortality rate in insulin-dependent Type I DM (T1DM) comparing to normoglycemia, while T2DM was not concluded as a significant risk factor.

In a retrospective database analysis of post-discharge outcomes following elective EVAR, Gupta et al. revealed that DM patients had higher overall post-discharge morbidity, mostly linked to higher wound infection rates (81). Another database-based retrospective study by Tarbunou et al. evaluated the association between post-operative hyperglycemia and prognosis following non-ruptured AAA repair (82). Approximately one in six patients undergoing elective AAA repair developed postoperative hyperglycemia and were associated with greater risk of infection and death and longer duration of hospital stay. Patients with hyperglycemia following EVAR had nearly 2 times the odds of infection and 7.5 times the odds of in-hospital mortality. Hyperglycemic patients underwent OSR had 3 times the odds of in-hospital mortality (82).

A prospective study enrolling 66 patients undergoing elective AAA repair reported DM defined by HbA1c ≥ 6.5% as an independent risk factor for mortality (83). Huang et al. conducted a meta-analysis that comprised 12 cohort studies involving a total 20,210 patients following AAA repair. A significantly higher long-term mortality was reported in diabetic patients comparing to normoglycemic group (5). Another meta-analysis by De Rango et al. found similar result of lower long-term survival rates and higher complication rates at 2-5 years following AAA repair among diabetic patients. This study also indicated an augmented 30-day/in-hospital operative mortality after AAA repair in DM patients (84). Based on these poor prognosis outcomes of DM following AAA repair, more attention should be paid to DM and related complications post AAA operations.

## 6 Conclusion

Although contribution of DM to AAA risk factors such as CAD and PAD have been well-established, an inverse relationship was revealed between DM and AAA. DM and hypoglycemic medications were found to target pathological hallmarks of AAA, thereby decreasing AAA incidence and prevalence rate, limiting AAA growth and preventing AAA rupture. Despite the protective effect of DM on AAA formation and progression, presence of DM or post-operative hyperglycemia was still found related to worse prognosis and higher long-term mortality rate post AAA repair. The double-edged nature of DM in AAA warranted further well-designed, prospective clinical investigations to formulate the standard of care for patients with AAA and DM, and to discover novel pharmacological target.

## Author contributions

Literature research: ZH and HS; Writing – original draft: ZH and HS; Writing – review and editing, TZ and YL. All authors contributed to the article and approved the submitted version.
